# Research Progress in Improving the Cycling Stability of High-Voltage LiNi_0.5_Mn_1.5_O_4_ Cathode in Lithium-Ion Battery

**DOI:** 10.1007/s40820-016-0123-3

**Published:** 2017-01-04

**Authors:** XiaoLong Xu, SiXu Deng, Hao Wang, JingBing Liu, Hui Yan

**Affiliations:** The College of Materials Science and Engineering, Beijing University of Technology, Beijing, 100124 People’s Republic of China

**Keywords:** High-voltage cathode, LiNi_0.5_Mn_1.5_O_4_, Lithium-ion battery, Cycling stability, Platform voltage

## Abstract

High-voltage lithium-ion batteries (HVLIBs) are considered as promising devices of energy storage for electric vehicle, hybrid electric vehicle, and other high-power equipment. HVLIBs require their own platform voltages to be higher than 4.5 V on charge. Lithium nickel manganese spinel LiNi_0.5_Mn_1.5_O_4_ (LNMO) cathode is the most promising candidate among the 5 V cathode materials for HVLIBs due to its flat plateau at 4.7 V. However, the degradation of cyclic performance is very serious when LNMO cathode operates over 4.2 V. In this review, we summarize some methods for enhancing the cycling stability of LNMO cathodes in lithium-ion batteries, including doping, cathode surface coating, electrolyte modifying, and other methods. We also discuss the advantages and disadvantages of different methods.

## Introduction

Although a commercial success, lithium-ion batteries (LIBs) are still the object of intense research mainly aimed to improve energy density for the requirement of electric vehicles (EVs), hybrid electric vehicles (HEVs), and smart grids [1–3]. High-voltage lithium-ion batteries (HVLIBs) with moderate theoretical discharge capacity, high thermodynamic stability, and stable high discharge platform offer new possibilities for next batteries with high energy density [4–6]. In the past research, polyanionic cathode materials [such as olivine LiMPO_4_ and monoclinic Li_3_M_2_(PO_4_)_3_] [7–9], borates (LiMBO_3_) [10], tavorite fluorosulphates (LiMSO_4_F) [11], and orthosilicates (Li_2_MSiO_4_) [12] were investigated. However, the lower discharge plateau leads to lower energy density.

The high-voltage LiNi_0.5_Mn_1.5_O_4_ (LNMO) cathode is the most promising candidate among the 5 V cathode materials for LIBs due to its flat plateau at 4.7 V [13], large specific capacity (146.6 mAh g^−1^), and a two-electron process Ni^2+^/Ni^4+^, where the Mn^4+^ ions remain electrochemically inactive [14, 15]. However, the degradation of cyclic performance is very serious when LNMO operates over 4.2 V. As a kind of HVLIB cathode material, LNMO was widely investigated and systematically reviewed. In 2011, Yi et al. [16] reported the developments in the doping of LNMO cathode material for 5 V LIBs, in which the rate capability, rate performance, and cyclic life of various doped LNMO materials were described. In 2013, Hu et al. [17] summarized the progress in high-voltage cathode materials and corresponding matched electrolytes, in which they introduced LNMO as high-voltage cathode materials. In 2015, Wang [18] devoted to tackle the difficulties of poor cyclic performance at high current densities and instability with electrolyte and reviewed the challenges and developments of LNMO-based compounds. Recently, Zhu et al. [19] highlighted the advancements in the development of advanced electrolytes for improving the cycling stability and rate capacity of LNMO-based batteries. We can find the developments of LNMO and researchers’ interest from recent reviews reports. However, these reviews only summarized the advantages of LNMO as the HVLIBs cathode, the modification methods of doping or electrolytes, etc. It is necessary to compare different modification methods based on the architectural features and cyclic degradation mechanisms of LNMO and find an effective method to improve the cycle performance of LNMO. In this review, focus is given to the approaches to improve the cycling stability of LNMO based on the synthesis of highly purified LNMO, structural reversibility of $Fd\bar{3}m,$ and cycling degradation mechanism of undesired reactions between LNMO and electrolyte.

## Synthesis, Structure, and Cycling Degradation Mechanism of LNMO

### Synthesis

The synthetic method of LNMO mainly includes dry synthesis and wet synthesis. Solid-state method is the most common method in which stoichiometric mixture of starting materials is ground or ball-milled together and the resultant mixture is heat-treated in a furnace [20, 21]. Wet synthesis, such as sol–gel method and co-precipitation method, are easy to control the size, morphology, and uniformity of the particles [22–25]. In this method, the purity of the material depends on the starting materials, calcination temperature, and time. It is mentioned that the resultant products from these methods generally contain impurity phases such as NiO [26, 27] and Li_*x*_Ni_1−*x*_O [28, 29] due to the oxygen loss at high temperature, which could lead to electrochemical deterioration and capacity fading.

In order to solve the problem of phase purity, molten salt method is a promising and simple technique. Highly pure LNMO materials have been prepared at relatively low temperatures taking advantage of the relatively higher diffusion rates between reaction components [30, 31]. In 2004, Kim et al. [32] synthesized highly pure LNMO through a modified KCl molten salt method using a mixture of LiCl and LiOH salts. It delivered an initial discharge capacity of 139 mAh g^−1^ with excellent capacity retention rate more than 99% after 50 cycles. Deng et al. [33] synthesized double-shell LNMO hollow microspheres without rock-salt impurity phase via a facile molten salt method. The capacity of LNMO remained about 98.3% after 100 cycles (116.7 mAh g^−1^ at 0.5 C between 3.5 and 5.0 V).

The molten salt method is based on the application of a salt with a low melting point. In the molten salt, diffusion rates between reaction materials are much higher, and thus powders with a single phase can be obtained at a lower temperature. Molten salt method is an effective approach in the synthesis of highly pure LNMO.

### Structure

**Fig. 1:**
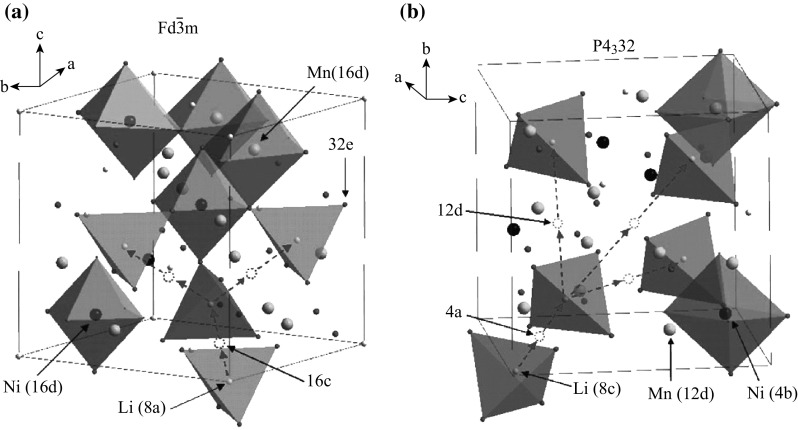
**a** A schematic view of face-centered cubic (FCC, $Fd\bar{3}m$) and **b** primitive simple cubic (SC, *P*4_3_32) structure [17]

As a promising cathode candidate for application in HVLIBs, LNMO has its own special crystal structure. It has two kinds of spinel crystal structures, face-centered cubic (FCC, $Fd\bar{3}m$), and primitive simple cubic (SC, *P*4_3_32) structures. For the FCC structure, the unit cell consists of the Li-ion-occupied tetrahedral 8a sites, Mn/Ni-ion-occupied octahedral 16d sites, and O-occupied cubic close packed 32e sites. The Mn/Ni ions in 16d sites are randomly distributed (Fig. 1a). For the primitive SC structure, the Li ions are located in the 8a sites, Mn ions in the 12d sites, Ni ions in the 4b sites, and oxygen ions in the 24e and 8c sites (Fig. 1b) [34, 35]. The crystal structures of FCC and SC are dependent on the annealing temperature in synthesizing process. SC spinel with a space group *P*4_3_32 is generally formed at *T* ≤ 700 °C, while a FCC spinel with a space group $Fd\bar{3}m$ is usually formed at *T* ≥ 800 °C [36–38].

**Fig. 2:**
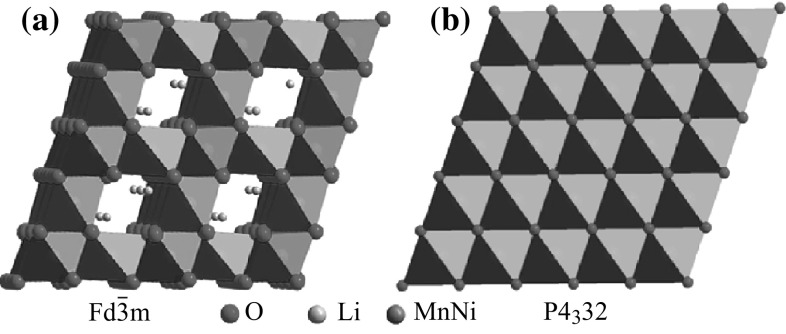
Schematic illustration of the Li^+^ migration paths during electrochemical reaction of both **a** $Fd\bar{3}m$- and **b** *P*4_3_32-structured LNMO [35]

$Fd\bar{3}m$ structure exhibits stable cycle ability compared to that of *P*4_3_32 structure because the *P*4_3_32 structure has a higher resistance than that of the $Fd\bar{3}m$ structure during delithiation. $Fd\bar{3}m$ structure undergoes a one-step phase transition, while *P*4_3_32 structure undergoes a two-step phase transition which is uncompleted. It is confirmed that LNMO with the space group of $Fd\bar{3}m$ has superior electrochemical behavior and structural reversibility compared to *P*4_3_32 [39–41]. Song et al. [35] described the differences of the Li^+^ migration paths during electrochemical reaction of both $Fd\bar{3}m$- and *P*4_3_32-structured LNMO (Fig. 2). Figure 2a shows obvious Li^+^ migration paths in the $Fd\bar{3}m$-structured LNMO, while there are no lithium-ion channels in the *P*4_3_32 structure (Fig. 2b). This comparison also suggested that the $Fd\bar{3}m$ has superior electrochemical behavior and structural reversibility compared to *P*4_3_32.

### Cycling Degradation Mechanism

**Fig. 3:**
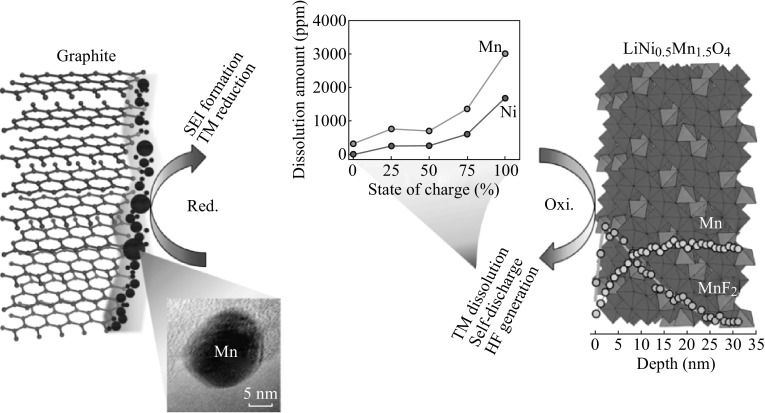
The cycling degradation mechanisms of high-voltage LNMO cathodes [59]

Charging the LNMO at high voltage (5 V) is proposed to be beneficial for its reversible capacity; however, it will accelerate the performance degradation. The failure mechanisms of HVLIBs were recently investigated [42]. It was found that electrode/electrolyte interface degradation, gas production, and transition metal dissolution are the leading factors. Charging the LIBs at high voltage can accelerate the oxidation of the electrolyte and result in the formation of a high impedance film on the electrodes surface. Furthermore, the formation of hydrofluoric acid (HF) at high voltage leads to a severe deterioration of the cycling performance [43–47]. The electrolyte reactions also result in gaseous products at higher potentials, which will cause pouch and prismatic cells to bulge [48–50]. Therefore, gas production is another failure mechanism that often occurs in lithium-ion cells at high voltage [51, 52]. In general, these gassing reactions can be attributed to electrolyte reactions on electrodes [53–55], and the gas products are H_2_, CO_2_, and low-weight hydrocarbons [56–58]. Figure 3 shows the dissolution behaviors of Mn and Ni in LNMO/graphite full cells at high voltage by Pieczonka [59]. It is found that the amounts of dissolved Mn and Ni, diethyl ether, as well as decomposition product of diethyl carbonate in electrolyte increase with state of charge, temperature, and storage time. The decomposition of electrolyte could be explained by the self-discharge behavior of LNMO, which promotes electrolyte oxidation. In addition, HF is believed to be generated during the formation of diethyl ether (via dehydration reaction from EtOH, and another decomposition product of diethyl carbonate), which can accelerate Mn and Ni dissolution from LNMO. Additional, various reaction products formed as a result of Mn and Ni dissolution, such as LiF, MnF_2_, NiF_2_, and polymerized organic species, were found on the surface of LNMO electrodes, which would increase battery-cell impedance. The specific mechanism is shown in Eqs. 1–4.

1 $${\text{LiPF}}_{6} + 4{\text{H}}_{2} {\text{O}} \to {\text{LiF}} + {\text{PO}}_{4} {\text{H}}_{3} + 5{\text{HF}}$$

2 $$2{\text{LiNi}}_{0.5} {\text{Mn}}_{1.5} {\text{O}}_{4} + 4{\text{H}}^{ + } + 4{\text{F}}^{ - } \to 3{\text{Ni}}_{0.25} {\text{Mn}}_{0.75} {\text{O}}_{2} + 0.25{\text{NiF}}_{2} + 0.75{\text{MnF}}_{2} + 2{\text{LiF}} + 2{\text{H}}_{2} {\text{O}}$$

3 $${\text{DEC}} + {\text{LiPF}}_{6} \to {\text{C}}_{2} {\text{H}}_{5} {\text{OCOOPF}}_{4} + {\text{C}}_{2} {\text{H}}_{4} + {\text{HF}} + {\text{LiF}}$$

4 $${\text{C}}_{2} {\text{H}}_{5} {\text{OCOOPF}}_{4} \to {\text{PF}}_{3} {\text{O}} + {\text{CO}}_{2} + {\text{C}}_{2} {\text{H}}_{4} + {\text{HF}}.$$

From the above-mentioned failure mechanisms, the cycle performance degradation of LNMO is mainly associated with the undesired reactions between electrodes and electrolyte. Therefore, the modifications of cathode materials and electrolytes are the key factors to improve the cycling stability of LNMO.

## Approaches to Improve the Cycling Stability of LNMO

### Doping

Doping is considered to be an effective way to modify the intrinsic properties of the electrode materials and to improve cycle performance of LNMO [60–62]. The commonly doping ions are metal cations and anions. These doping ions are able to improve the cycling stability by altering the crystal compositions, structures, and parameters of LNMO.

Theoretical studies predict that doping with transition metal would increase the capacity, whereas doping with non-transition metal would lead to increased voltage [63]. In the past, various elements were proposed by different research groups to impact the LNMO structure, electrical conductivity, stability on Li insertion/deinsertion, and capacity retention on cycling, e.g., Ti [60], Cr [64], Mn [65], Ni [66], Fe [61], Cu [67], Bi, Zr, Sn [62], Zn [63], Mo, and V [68]. It was found from the past research that doping mainly affected the surface morphology, phase compositions, and the crystal parameters of the LNMO cathode material particles. Schroeder et al. [69] reported that post-doping with titanium for the preparation of LiNi_0.5_Mn_1.47_Ti_0.03_O_4_ (LNMTO) led to nanocrystalline LNMTO granules with homogenous titanium distribution. These Ti-doped materials exhibited further increased specific capacity, specific energy, and cycling stability due to the reduced Mn^3+^ content and their particular microstructure.

**Fig. 4:**
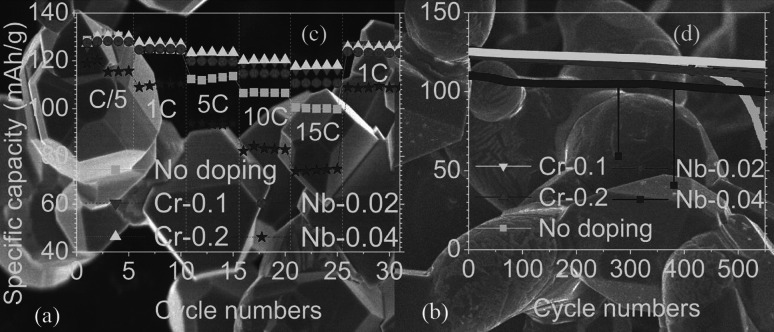
SEM images of **a** Cr doping and **b** Nb doping, **c** rate cycle performance and **d** cycle performance of all samples. Nb-0.02: LiNb_0.02_Ni_0.49_Mn_1.49_O_4_, Nb-0.04: LiNb_0.04_Ni_0.48_Mn_1.48_O_4_, Cr-0.1: LiCr_0.1_Ni_0.45_Mn_1.45_O_4_, Cr-0.2: LiCr_0.2_Ni_0.4_Mn_1.4_O_4_ [70]

Jing et al. [70] synthesized undoped, Cr-doped, and Nb-doped LNMO via a polyvinylpyrrolidone combustion method by calcinating at 1000 °C for 6 h. Scanning electron microscopy (SEM) images showed that Cr doping resulted in sharper edges and corners and smaller particle size (Fig. 4a), while Nb doping led to smoother edges and corners and more rounded and larger particles (Fig. 4b). Cr doping and light Nb doping improved the rate cycle performance of LNMO (Fig. 4c) due to the fact that Cr and light Nb doping speeded up Li^+^ diffusion and reduced the resistance of Li^+^ through the solid electrolyte interface (*R*_SEI_), the charge-transfer resistance of Li^+^, and electrons (*R*_ct_) of LNMO particles. The cycling performance was improved by Cr or Nb doping (Fig. 4d). The LiCr_0.1_Ni_0.45_Mn_1.45_O_4_ remained at 94.1% capacity after 500 cycles at 1 C, and during the cycling the coulombic efficiency and energy efficiency remained at over 99.7% and 97.5%, respectively.

**Fig. 5:**
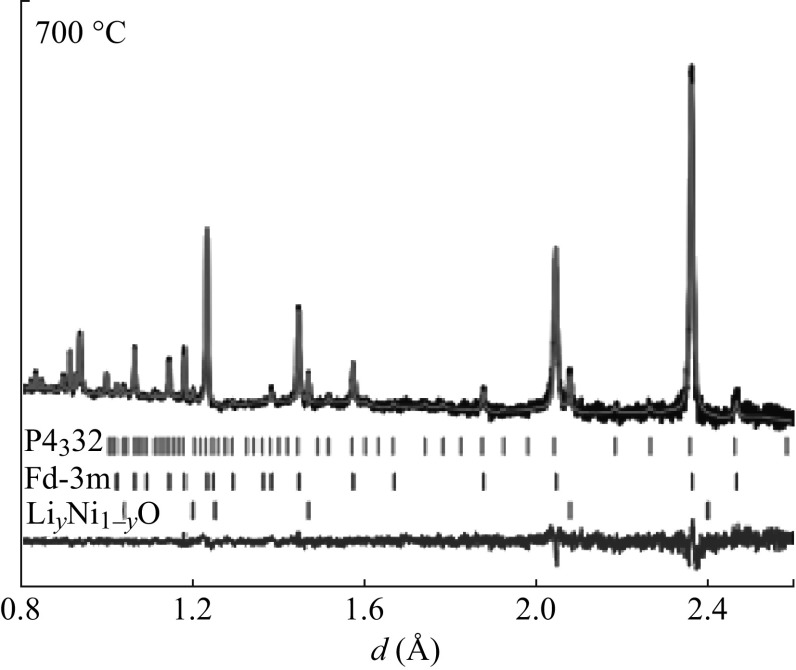
NPD patterns of the Cr-doped spinel prepared at 700 °C [71]

Kosova et al. [71] prepared the pure LNMO and doped spinels LiNi_0.5−*x*_Mn_1.5−*y*_M_*x*+*y*_O_4_ (M = Co, Cr, Ti; *x* + *y* = 0.05) by mechanochemically assisted solid-state synthesis. Compared with pure LNMO, the doped spinels at 700 and 800 °C showed high specific capacity and good cycle ability in 3.0–4.85 V. For all doped samples, the enlarged lattice parameter after doping (Table 1) was the main reason for the improvement in the electrochemical properties. Based on the neutron powder diffraction (NPD) data (Fig. 5; Table 1), the doped samples at 700 °C consist of predominantly $Fd\bar{3}m$ phase. The improvement in the electrochemical properties was attributed to LNMO with the space group of $Fd\bar{3}m$ ($Fd\bar{3}m$ has superior electrochemical behavior compared to *P*4_3_32).

**Table 1:** Refined lattice parameters of the undoped LNMO and doped LiNi_0.5−*x*_Mn_1.5−*y*_M_*x*+*y*_O_4_ (M = Co, Cr, Ti; *x* + *y* = 0.05) spinel from neutron powder diffraction (NPD) data [71]

Lattice parameter	Undoped spinel		Co ($R_{{{\text{Co}}^{ 3+ } }} = 0. 5 4 5$ Å)		Cr ($R_{{{\text{Cr}}^{3 + } }} = 0. 6 1 5$ Å)		Ti ($R_{{{\text{Ti}}^{4 + } }} = 0. 60 5$ Å)	
	700 °C	800 °C	700 °C	800 °C	700 °C	800 °C	700 °C	800 °C
*a* (Å)	8.1697 (3)	8.1710 (1)	8.1739 (3)	8.1762 (1)	8.1754 (3)	8.1784 (1)	8.1819 (3)	8.1849 (1)
*V* (Å^3^)	545.28 (5)	545.54 (2)	546.13 (3)	546.58 (2)	546.42 (3)	547.03 (2)	547.74 (3)	548.34 (2)
*Fd*−*3m*/*P*4_3_32/Li_*y*_Ni_1−*y*_O, ratio (%)	–/100/–	95.9/–/4.1	87.2/5.2/7.6	93.2/–/6.8	85.1/9.4/5.5	97.2/–/2.8	84.5/10.4/5.1	96.5/–/3.5
*Χ* ^2^	1.83	1.27	1.93	2.44	1.40	2.08	2.43	1.89
*R*_wp_ (%)	4.10	3.88	4.28	3.89	3.80	3.71	4.13	3.66

In addition to the metal doping, anions, such as F and S, are also effective for stabilizing the structure of spinel LNMO. F-doped samples show better resistance against HF attack than undoped samples. F-doping could suppress the formation of NiO impurity and simultaneously reduce the voltage polarization. Oh et al. [72] reported that F-doped LNMO cathodes synthesized by ultrasonic spray pyrolysis method exhibited superior structural properties and rate capability. Xu et al. [73] reported LiNi_0.5_Mn_1.5_O_3.975_F_0.05_ prepared by sol–gel technique reannealing in oxygen and LiF as fluorine source. The result showed that F-doping enhances the initial capacity from about 130 to 140 mAh g^−1^ between 3.5 and 5.2 V compared with undoped LNMO. Du et al. [74] reported F-doped LiNi_0.5_Mn_1.5_O_4−*x*_F_*x*_ (0.05 ≤ *x* ≤ 0.2) prepared by sol–gel and post-annealing treatment method. The compound LiNi_0.5_Mn_1.5_O_3.9_F_0.1_ displayed good electrochemical properties of an initial capacity of 122 mAh g^−1^ and a capacity retention of 91% after 100 cycles. The research results indicated that F-doping made spinel structure more stable due to the strong M-F bonding, which was favorable for the cyclic stability. Sun et al. [75] reported the LiNi_0.5_Mn_1.5_O_4−*x*_S_*x*_ (*x* = 0 and 0.05) synthesized by co-precipitation. The S-doped LNMO displayed excellent capacity retention and rate capability compared with undoped LNMO material. The enhanced electrochemical behavior of the S-doped spinel is attributed to the rough morphology of the primary particles with smaller particle size.

In addition, Lee [76], Nobili [77], and Rao [78] systematically investigated the effects of Al, Cu, Zr, and Ti elements doping on the cycle performances of LNMO cathode materials, respectively. Studies showed that the improvement of cycling stability of HVLIBs by doping was mainly attributed to the influences of doped ion on alterations of the crystal compositions, structures, and parameters.

### Cathode Surface Coating

Although the metal-ion doping is able to improve the cycling stability of LNMO, it could not fundamentally overcome the shortcomings of LIBs under high voltage because doping is unable to prevent the undesired side reactions between cathode and electrolyte. The protective surface modification is required in this case. The cathode surface modifications mainly include inorganic coating and organic coating.

#### Inorganic Coating

Inorganic materials are potential materials for modifying the particle surfaces and improving the electrochemical performances of LNMO with respect to the rate performance and cycling life. The main role of inorganic coating is preventing electrode reaction with the electrolyte and protecting cathodes from crystal destruction to some extent [79, 80]. Different inorganic materials have different advantages on the surface modifications of LNMO cathodes. The commonly used inorganic materials include metallic oxides (ZnO, Bi_2_O_3_, and Al_2_O_3_) [81–84], conventional cathode materials (LiNbO_3_, LiMn_2_O_4_, Li_4_Ti_5_O_12_, Li[Li_0.2_Mn_0.6_Ni_0.2_]O_2_, and LiFePO_4_) [85–89], and metal fluorides (LiF, MgF_2_, and AlF_3_) [90–92].

Coating cathode materials with metallic oxides are able to significantly improve the cycle performances of LNMO. This is attributed to the fact that the surface coating of cathode materials can cut off the cathode contact with the electrolyte and suppress the dissolution of active substances. Fan et al. [93] investigated the morphology, structures, and performances of the SiO_2_-coated LNMO cathode materials for HVLIBs. The results indicate that the surfaces of the coated LNMO samples were covered with porous, amorphous, nanostructured SiO_2_ layers and the capacity retention rates were obviously improved. Lee et al. [94] utilized SnO_2_ coating to modify LNMO cathode by employing electron cyclotron resonance metal–organic chemical vapor deposition and a conventional tape-casting method. The SnO_2_-deposited LNMO electrodes exhibit superior electrochemical performances during the storage test in a fully charged state than the pristine LNMO electrode. Wang et al. [4] synthesized V_2_O_5_-coated LNMO cathode materials via a wet-coating method. High-resolution transmission electron microscopy (HRTEM) images showed clear lattice fringes of all LNMO samples, and the V_2_O_5_ coating layer was about 3 nm in 5% V_2_O_5_-LNMO sample. The selected area electron diffraction pattern (SAED) suggested that the LNMO sample was of ordered lattice and single-crystal structure. The cycling performances profiles of different materials showed that the 5% V_2_O_5_-LNMO sample had the best performance. V_2_O_5_ as a protective layer inhibited the electrolyte decomposition at the electrode/electrolyte interface, offered a 2D path for Li^+^ diffusion, and reduced metal-ion dissolution, thereby improving the structure integrity and capacity retention during charge/discharge cycles.

**Fig. 6:**
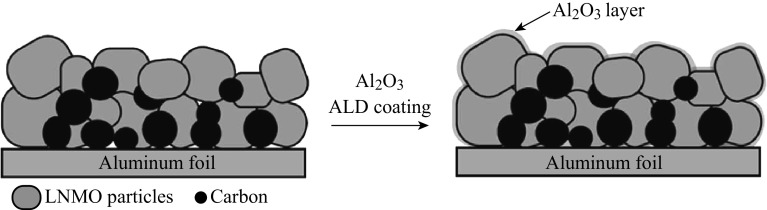
Schematic of ALD process on LNMO electrode composite [96]

The coating thickness was determined by a tradeoff between a high Li^+^ permeability and Mn-ion impermeability. In this regard, the coating uniformity is an important requirement because extra-thick areas would compromise the cathode performance and the extra-slim areas would compromise the coating protective ability. Atomic layer deposition (ALD) is an effective technique to achieve uniform coating on the surface of LNMO materials. The ultrathin layer which is synthesized by ALD could suppress the undesirable reactions during cycling while retain the electron and ion conductivity of the electrode. The Al_2_O_3_ layer comes from 30 cycles ALD coating, and the thickness of Al_2_O_3_ is 3–4 nm. Between 3.5 and 5.0 V, the Al_2_O_3_-coated LNMO still delivers 116 mAh g^−1^ at the 100th cycle; in comparison, the capacity for bare LNMO decreases to 98 mAh g^−1^. The Al_2_O_3_-coated LNMO retains 63% of its capacity after 900 cycles at 0.5 C [95]. Figure 6 shows the model of surface modification by taking advantage of ultrathin layers of Al_2_O_3_ by ALD to protect the LNMO cathode from undesired side reactions at its electrode/electrolyte interface [96].

Coating with conventional LIB cathode material is an effective method to improve the cycle performances of LNMO cathodes. LiFePO_4_ (LFP) is a promising surface coating material due to its thermal stability and low cost. Nanosized LFP with appropriate amount of carbon coating exhibits high-rate performances as well as long cycling life [97, 98], such as LFP-coated LiCoO_2_ [89] and LFP-coated Li[Ni_0.5_Co_0.2_Mn_0.3_]O_2_ [99]. LFP also is a superior coating material for LNMO cathodes.

**Fig. 7:**
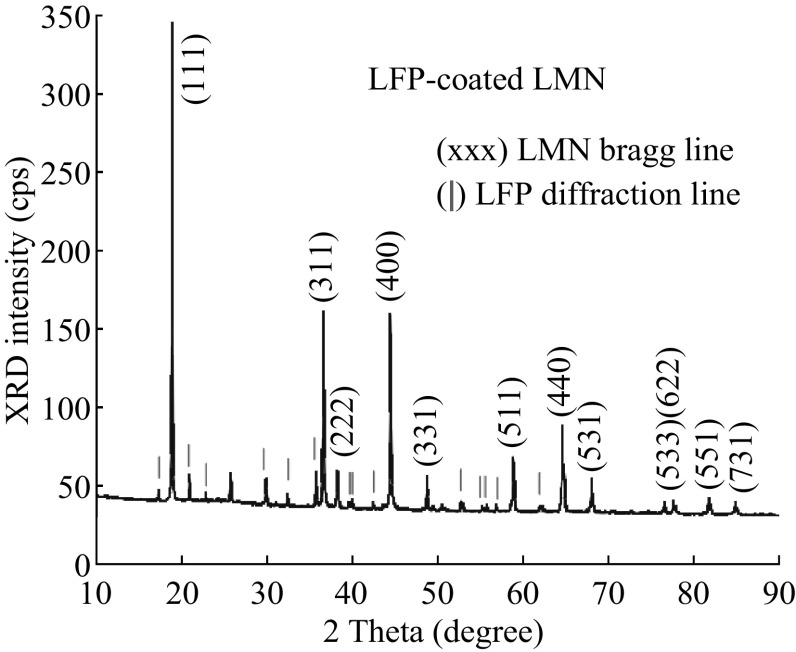
**a** XRD pattern of the C-LFP-coated LNMO sample. The Bragg lines indexed are those of the spinel LNMO lattice, while the main lines of the LiFePO_4_ olivine are marked in red [100]

The LFP-coated LNMO was synthesized by a mechano-fusion dry process. Commercial LFP served as guest particles and LNMO served as host particles, which was directly dry milled for several minutes in a Mechano Fusion System with the mass ratio of 1:4 [100]. The results of the X-ray diffraction (XRD) diagram suggested that there was no structural change after mechano-fusion dry process (Fig. 7). After the mechano-fusion, the XRD spectrum simply contained the additional peaks associated to the LFP part (red marks in Fig. 7), which indicated that the LFP coating layer was well crystallized. The discharge capacity of pristine LNMO decreased from 105 to 65 mAh g^−1^ after 100 cycles 1 C rate, and the capacity retention ratio was only 61.5%. In contrast, LFP-coated LNMO delivered a capacity of 82 mAh g^−1^ with capacity retention ratio of 74.5% after 140 cycles. Improved cycling stability of the LFP-coated LNMO was attributed to the fact that the LFP coating prevented the LNMO particles from the undesired side reactions with electrolyte. Moreover, the carbon-LFP layer could also increase the conductivity of the cathode.

The other simple solution was to employ a metal fluoride coating, which was able to be stable against HF attack. Up to now, a number of works were focused on the preparation and investigation of cathode materials with fluoride coating and different fluorides were evaluated: LiF [101], SrF_2_ [102, 103], MgF_2_ [104, 105], CaF_2_ [106, 107], AlF_3_ [108, 109], GaF_3_ [110], CeF_3_ [111], and LaF_3_ [112, 113].

The improvement of cycling stability was mainly attributed to the “buffer” layer provided by the AlF_3_ coating, through which the extracted oxygen was reduced in its activity and suppressed the electrolyte decomposition at high voltages [114]. Li et al. [115] reported that the AlF_3_-coated LNMO samples showed better rate capability and higher capacity retention than the uncoated samples. Among these samples, 4.0 mol% coated sample exhibited the highest cycling stability. The 40th cycle discharge capacity at 300 mA g^−1^ current still remained 114.8 mAh g^−1^, while only 84.3 mAh g^−1^ for the uncoated sample.

**Fig. 8:**
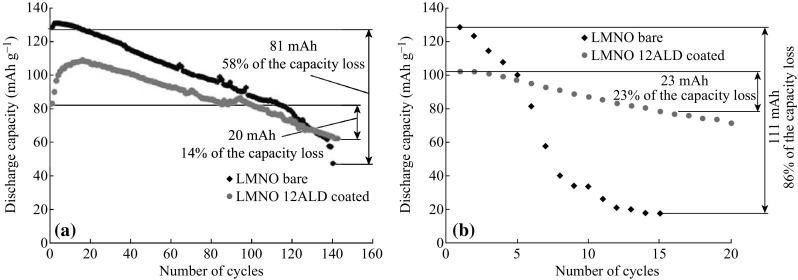
Capacity versus cycle number for bare LMNO material and ALD-coated LMNO material (12 ALD-layer coating, C/10 rate): **a** room temperature, **b** 45 °C [116]

Kraytsberg et al. [116] successfully deposited a several atomic layer thick uniform magnesium fluoride film onto LNMO powders by ALD techniques. Whereas the film moderately diminished initial cathode performance, it substantially extended the cycle life of the LNMO cathodes. The protective effect was particularly pronounced at 45 °C (Fig. 8). It was suggested that the cycling improvements was because the ALD film prevented the access of the aggressive byproducts of electrolyte decomposition (particularly HF) to the LNMO surface. Huang et al. [110] prepared GaF_3_-coated LNMO materials. The 0.5 wt% GaF_3_-coated LNMO delivered a discharge capacity of 97 mAh g^−1^ at 20 °C, while the pristine sample only yielded 80 mAh g^−1^ at 10 °C. Meanwhile, the 0.5 wt% GaF_3_-coated LNMO exhibited an obviously better cycle life than the bare sample at 60 °C, delivering a discharge capacity of 120.4 mAh g^−1^ after 300 cycles, 82.9% of its initial discharge capacity, while the pristine only gave 75 mAh g^−1^. The improvements were attributed to the fact that the GaF_3_ layer not only increased the electronic conductivity of the LNMO but also effectively suppressed the undesired reaction between the LNMO and the electrolytes, which reduced the charge-transfer impedance and the dissolution of Ni and Mn during cycling.

#### Organic Coating

Surface modification with inorganic materials such as metallic oxides, metal fluorides, and cathode materials focused on how to control interfacial side reaction between LNMO and liquid electrolyte at high voltages. Unfortunately, the inorganic materials tend to be discontinuously deposited onto the LNMO surface, and would also act as an inert layer regarding ionic conduction. Moreover, the inorganic coatings often require complex and cost-consuming processing steps. On the other hand, surface modification with organic materials such as polyimide (PI) and polypyrrole (PPy) are able to solve the problems of discontinuously deposition, complex processing steps, and high cost.

Recently, PI encapsulation generated from polyamic acid (PAA) was reported to improve the cyclic stability of LiCoO_2_ and LiNi_1/3_Mn_1/3_Co_1/3_O_2_ [117–119]. The effects of surface modifications with PI [82, 120–123] were reported and showed improvements in the performances of LNMO cathodes, too. The high polarity and outstanding film forming capability of PAA, plus its strong affinity to transitional inorganic materials surfaces, might contribute to a facile formation of a nanometer thick, highly continuous, and ionic-conductive PI encapsulating layer on the surface of active materials [124]. Particularly, Kim et al. [125] reported that the LNMO cathodes modified by PI coating presented excellent cycling stability with capacity retention of >90% after 60 galvanostatic cycles at 55 °C.

**Fig. 9:**
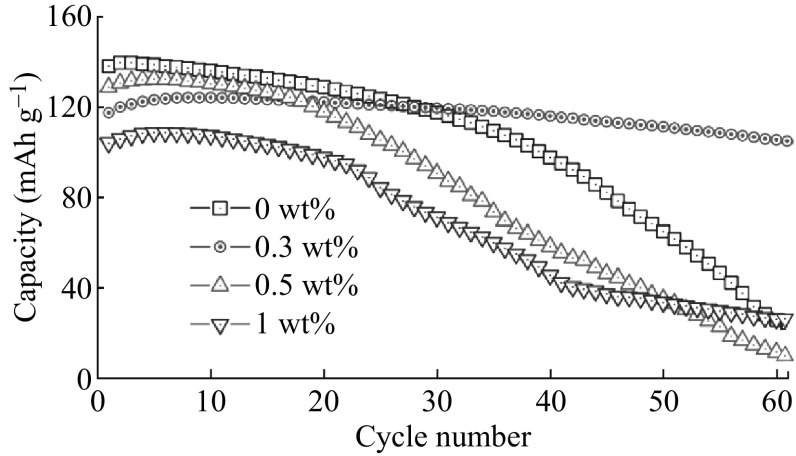
Galvanostatic cycle profiles of spinel phase LNMO cathodes with various concentrations of polyimide (PI) coating in half-cell assembly tested at 3.5–5 V versus Li and a current density of 0.2 mA cm^−2^ at 55 °C [125]

Kim et al. [125] presented the influences of PI coating concentration on the electrochemical properties of LNMO cathodes, particularly under elevated temperature conditions. All test cells delivered good cycle ability under ambient temperature conditions, irrespective of the PI coating concentration, with a prominent plateau at 4.7 V versus Li, whereas all test cells experienced the poorest electrochemical behavior under elevated temperature conditions except 0.3 wt% PI. The 0.3 wt% PI coated LNMO phase delivered excellent cycle ability with capacity retention of >90% at 55 °C (Fig. 9). In comparison to conventional inorganic material coatings, distinctive features of the unusual PI wrapping layer were the highly continuous surface coverage with nanometre thickness (10 nm) and the provision of facile ion transport, which was reported by Cho et al. [126]. The nanostructure-tuned PI wrapping layer served as an ion conductive protection skin to suppress the undesired interfacial side reaction, effectively prevented the direct exposure of the LNMO surface to liquid electrolyte. As a result, the PI wrapping layer played a crucial role in improving the high-voltage cell performance and alleviating the interfacial exothermic reaction between charged LNMO and liquid electrolyte. However, the rate capability was not sufficiently improved due to the poor conductivity of PI.

PPy attracted increasing attention over the past decades because of their remarkable electrical conductivity, good electrocatalytic properties, cost-effective processability, lightweight, tunable mechanical and magnetic properties, and environmental friendliness [127]. They were explored for versatile applications, for examples, electrocatalysts [128], anticorrosion coatings [129], carbon dioxide captures [130], batteries [131], and electrochemical capacitors [132]. Compared with PI, organic material PPy was a typical cathode coating materials due to its good mechanical flexibility, chemical stability, and theoretical capacity of 72 mAh g^−1^ in LIBs [133]. In order to improve electrochemical performances of electrodes, PPy was used for Fe_3_O_4_/PPy [133], Fe_2_O_3_/PPy [134], LiMn_2_O_4_/PPy [135], LiV_3_O_8_/PPy [136, 137], LiFeO_2_/PPy [138], LiFePO_4_/PPy [139], LiNi_1/3_Co_1/3_Mn_1/3_O_2_/PPy [140], etc.

**Fig. 10:**
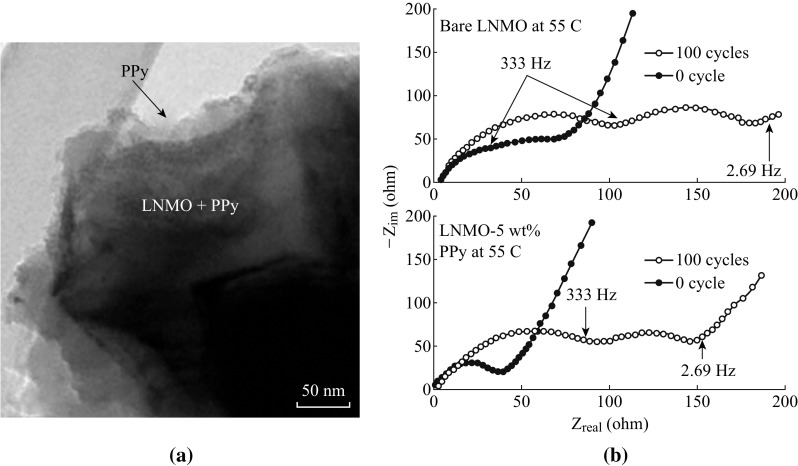
**a** TEM images of the LNMO-5 wt% PPy. **b** Nyquist plots of pristine LNMO and LNMO-5 wt% PPy electrodes before cycling and after cycling at 55 °C [141]

Gao et al. [141] investigated the PPy-coated LNMO spinel. The bare LNMO delivered a discharge capacity of 116 mAh g^−1^ at the first cycle. After that, the discharge capacity continuously decreased and only 76.7% capacity retention was achieved after 300 cycles. In contrast, capacity retentions of 83.2, 91.0, and 85.7% were obtained for composites with 3, 5, and 8% PPy over 300 cycles, respectively. The reversible capacities were, respectively, 105, 98, 92, and 85 mAh g^−1^ at 2.0, 3.0, 4.0, and 5.0 C. When the rate returned to 1.5 C, the specific capacity recovered up to 117 mAh g^−1^, indicating a very stable cycling performance. The uniform PPy coating on the surface of the LNMO (Fig. 10a) not only acted as an ion conductive layer but also suppressed the decomposition of Mn and Ni at high voltage. Two condensed semicircles were observed in the spectrum of the bare LNMO electrode at 55 °C before cycling (Fig. 10b), which indicated that a small portion of the electrolyte was decomposed and was directly deposited on the surface of the electrode after storage at high temperature. The electrolyte decomposition already formed a SEI layer on the active material before cycling. In contrast, the LNMO-5 wt% PPy cell only showed one semicircle with a diameter of 42 Ω, indicating a faster interfacial charge transfer.

**Fig. 11:**
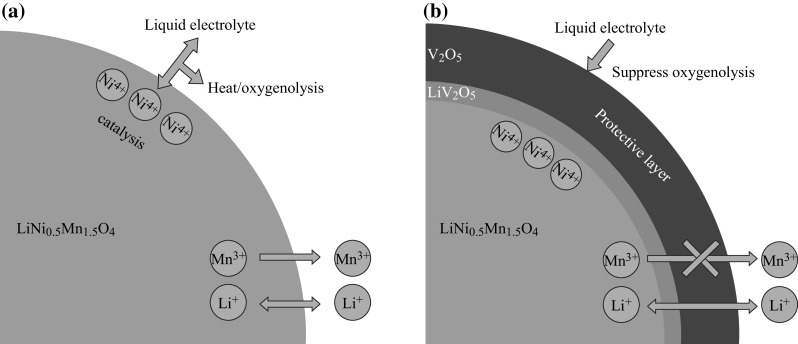
Schematic illustrations of the coating layer to suppress the unfavorable interfacial side reactions between coating layer and electrolyte [4]

Inorganic coatings and organic coatings have similar roles in the improvements of cycle ability for LNMO cathodes. Figure 11 illustrates the working mechanism of protective layer. The protective layers inhibited the electrolyte decomposition at the electrode/electrolyte interface, offered paths for Li^+^ diffusion, and reduced Mn^3+^ metal-ion dissolution, thereby improving the structure integrity and capacity retention during charge/discharge cycles. Compared with inorganic coating, the high polarity and outstanding film forming capability of organic coating, plus its strong affinity to transitional inorganic materials surfaces, might contribute to a facile formation of a nanometer-thick and highly continuous encapsulating layer on the surface of active materials. Compared with PI, PPy is more suitable for coating on surface of LNMO cathodes due to its remarkable electrical conductivity, lightweight, environmental friendliness, good mechanical flexibility, chemical stability, and theoretical capacity of 72 mAh g^−1^ in LIBs.

### Electrolyte Modifying

Surface coating is an effective method to improve the cycling stability of LNMO cathodes. However, it is difficult to extend for large-scale battery applications due to the material modification through complicated synthetic procedures. The surface coating improves the cycle ability but would reduce the discharge capacity of the high-voltage materials. Furthermore, the conventional LIBs employ organic carbonate esters as the electrolyte solvent, in particular, mixtures of ethylene carbonate (EC) with dimethyl carbonate (DMC), diethyl carbonate (DEC), and ethyl methyl carbonate (EMC) dissolved in LiPF_6_ salt. This electrolyte continuously decomposes above 4.5 V versus Li^+^/Li, limiting its application to a cathode chemistry that delivers capacity at a high charging voltage [142, 143]. Under the circumstances, the demand for a high-voltage electrolyte becomes a high priority for the development of LIBs with high ED, such as solid electrolyte, fluorinated electrolytes, as well as electrolyte additives.

#### Solid Electrolyte

It is well known that many solid electrolytes have a voltage window beyond 5 V and thus do not decompose under anodic current, such as Li_10_GeP_2_S_12_ [144], Li_3_PS_4_ [145], Li_4_SnS_4_ [146], Li_7_La_3_Zr_2_O_12_ [147], and lithium phosphorus oxynitride (Lipon) [148]. Furthermore, with a solid electrolyte, the concern of transition metal dissolution into the electrolyte is minimal. Compared with carbonate electrolytes, most ceramic solid electrolytes are intrinsically non-flammable. Lastly, lithium metal is compatible with many solid electrolytes and is less likely to form dendrites during cycling because of the mechanical robustness of the solid electrolyte [149].

**Fig. 12:**
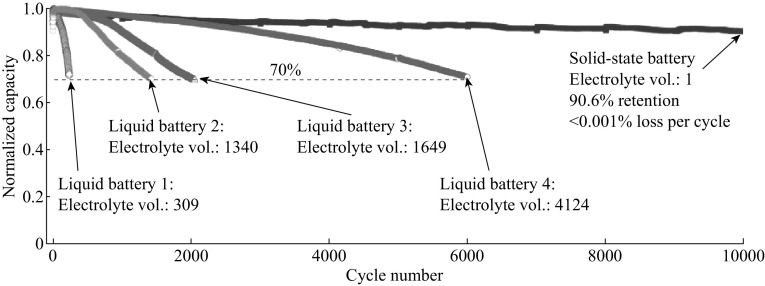
Capacity retention of high-voltage solid-state and liquid-electrolyte lithium batteries. The cathode is LNMO cathode, and the anode is Li metal. Volume of electrolyte was normalized to the volume of the cathode. All cells were cycled under a rate of 5 C. Volume of the cathode:electrolyte are 1:309, 1:1340, 1:1649, and 1:4124 for Liquid battery 1–4, respectively. Solid-state battery electrolyte vol.: (volume of the cathode:electrolyte = 1:1) [152]

Among all the solid electrolytes, Lipon is used as the model solid electrolyte mainly because of its wide voltage window (0–5.5 V) [148] and excellent interfacial compatibility with both cathodes and anodes [148, 150]. Fabrication of thin-film battery with LNMO cathode is challenging [151], but the model solid electrolyte in the performances is successfully applied. Li et al. [152] demonstrated that the solid-state HVLIB (the solid-state high-voltage battery consists of LNMO cathode, Lipon electrolyte, and Li metal anode) delivered outstanding cycling performance with 90% capacity retention and high coulombic efficiency of 99.98% after 10,000 cycles between 5.1 and 3.5 V at 5 C (Fig. 12), while the amount of electrolyte was thousands of times less than that in liquid-electrolyte batteries. The solid-state system enabled the full utilization of HVLIB by solving all problems associated with conventional batteries using liquid electrolyte: unstable electrolyte, dissolution of transition metals from the cathode, serious safety issues associated with the flammability of the electrolyte, and the roughening of the Li metal anode. Unfortunately, the prominent problem of solid-state batteries is their low power densities compared with liquid-electrolyte lithium batteries, resulting from the low ionic conductivity of the solid electrolyte, the electrode/electrolyte interfacial compatibility, and limited kinetics of the electrodes [153, 154]. On the other hand, interfacial instability between the electrode and electrolyte is a great challenge for solid-state batteries [155, 156].

Solid electrolytes are able to provide advantages over liquid electrolytes in terms of safety, reliability, and simplicity of design, but the ionic conductivity of solid electrolytes are generally lower than those of liquid electrolytes. Although some solid electrolytes have the highest conductivity, they have some disadvantages over other potential electrolytes, such as in mechanical strength or electrode compatibility. It is necessary to select a suitable solid electrolyte for a particular battery application based on the factors of operating parameters (such as voltage range and temperature) and battery design (such as rigid and flexible).

#### Fluorinated Electrolytes

Due to their high conductivity, excellent solubility with lithium salts, and ability to form a stable SEI, carbonates are still an excellent choice as the solvent for electrolyte systems [157]. However, traditional carbonates like EC and EMC have low potential limits, which make them unstable in high-voltage cells. Fluorinated organic solvents were investigated for many applications in LIBs due to their higher oxidation potential according to a density functional theory calculation [158]. Compared with the conventional electrolytes based on non-fluorinated solvents, fluorinated solvents might bring a variety of benefits to the electrolyte. For example, fluorinated cyclic carbonate was used as a co-solvent [159, 160] or as a SEI formation additive for graphite and silicon anodes [161]. A series of fluorinated linear carbonates were designed and synthesized as new electrolyte components to improve the low-temperature performance of LIB for deep space applications [162, 163]. And there are some studies about the performances of these fluorinated solvents for LNMO cathodes. Fluorinated molecules have higher oxidation potentials than their non-fluorinated counterparts due to the strong electron-withdrawing effect of the fluorine atom.

**Fig. 13:**
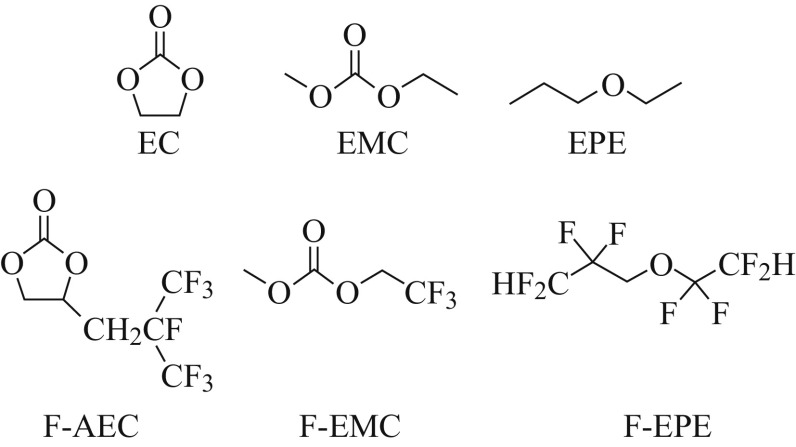
Chemical structure of the baseline carbonate (EC and EMC), ethyl propyl ether (EPE), fluorinated cyclic carbonate (F-AEC), fluorinated linear carbonate (F-EMC), and fluorinated ether (F-EPE) [164]

As shown in Fig. 13, Zhang et al. [164] depicted the chemical structures of the conventional electrolyte solvents (EC and EMC), ether (EPE), fluorinated carbonates (fluorinated cyclic carbonate, F-AEC and fluorinated linear carbonate, F-EMC), and fluorinated ether (F-EPE). The electrolytes composed of F-AEC, F-EMC, and F-EPE have superior stability compared with the EC/EMC-based electrolyte. The electrochemical floating test shows that the substitution of EMC with F-EMC and EC with F-AEC greatly improves the voltage limits of the electrolyte in HVLIBs at elevated temperatures. The fluorinated electrolytes have higher reduction potential, resulting in stability on the anode side of the graphite/LNMO cells [165]. Hu et al. [163] evaluated the fluoroethylene carbonate (FEC)-based high-voltage electrolyte for the LNMO/graphite by comparison with the conventional EC-based electrolyte. The high-voltage electrolyte showed much better cycle performance at both room temperature and 55 °C. In the post-test analysis, SEM and Fourier-transform infrared analyzes (FTIR) of the harvested anode and cathode cycled under 55 °C indicated that the high-voltage electrolyte had less solid decomposition products deposited on both the anode and the cathode. This finding is a direct proof of the enhanced stability of the high-voltage electrolyte in the LNMO/graphite system on both the LNMO cathode and graphite anode at high-temperature cycling conditions.

#### Electrolyte Additives

Some functional additives could be electrochemically polymerized prior to the electrolyte solvent decomposition to form a protective layer of conducting polymer film on the electrode surface. In order to suppress the reaction between the LNMO and electrolytes in HVLIBs, several electrolyte additives were so far identified to be suitable for LNMO cathodes, including inter alia tris (hexafluoro-iso-propyl) phosphate [166], lithium bis(oxalate) borate [167], 1,3-propane sultone [168], thiophene derivatives [169], *N*,*N*′-4,4′-diphenylmethane-bismaleimide [170], 1-propylphosphonic acid cyclic anhydride [171], trimethylboroxine [172], and glutaric anhydride [173]. These organic additives were electrochemically polymerized more quickly than the base electrolyte solution during charging batteries and tended to form a conductive film on the cathode at high voltages, then suppressed the decomposition of electrolyte solvents, and improved the cycling performances of the batteries [174–176].

Lee et al. [177] reported an improved cycling stability and reduced swelling behavior for a LNMO/graphite lithium-ion full cell using a combination of 1,3-propane sultone and succinic anhydride, a derivative of glutaric anhydride, as electrolyte additives. They concluded that the improved cycling stability would originate from the SEI forming ability of these additives on the surface of electrodes and their electrochemical stability on the cathode toward high potentials. Therefore, the formation of protective films through using the reducible and oxidative additives in the electrolytes was one of the most effective and easiest strategies to stabilize the interface of electrode–electrolyte.

The cycling performance of Li/LNMO cells with 1.0 M LiPF_6_ in EC/EMC (3/7) with and without added dimethyl methylphosphonate (DMMP) (0.5–1.0%) were investigated by Xu et al. [178]. Addition of DMMP resulted in improved capacity retention during cycling at 4.9 V versus Li. Ex-situ surface analysis of LNMO electrodes after cycling via SEM, X-ray photoelectron spectroscopy (XPS), and FTIR suggested that addition of DMMP inhibited electrolyte decomposition on the surface of the cathode. Addition of DMMP also inhibited the dissolution of Mn from LNMO particles stored in electrolyte at 85 °C.

**Fig. 14:**
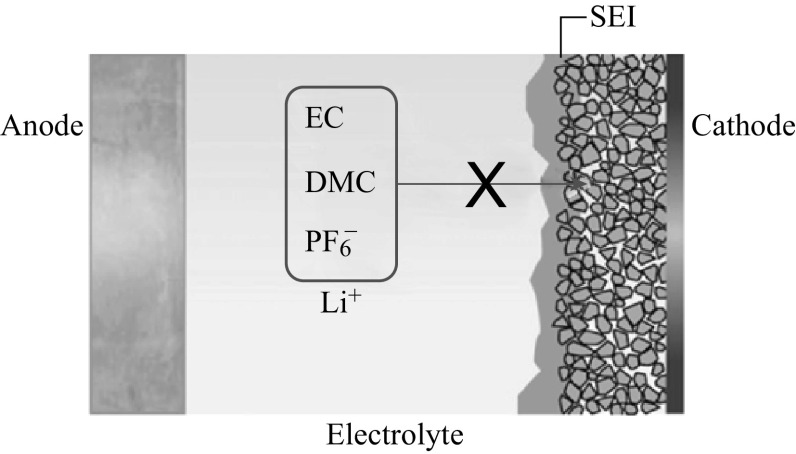
Schematic illustration of a protective SEI layer on the cathode surface, inhibiting further oxidative electrolyte decomposition and thus, lithium re-insertion [179]

Tarnopolskiy et al. [179] reported that succinic anhydride appeared to have a beneficial effect, lowering significantly the concomitant potential decay and capacity loss by up to more than 50%, while at the same time improving the coulombic efficiency and reducing the capacity loss per cycle. SEM and XPS analysis of cycled electrodes revealed a modified LNMO particles surface and the formation of a thinner, more stable SEI film on galvanostatically cycled electrodes by adding succinic anhydride to the base electrolyte. Succinic anhydride as suitable electrolyte additives formed a protective SEI layer on the cathode surface, inhibiting further oxidative electrolyte decomposition and thus, lithium re-insertion (Fig. 14). The most promising and efficient strategy for inhibiting the continuous electrolyte decomposition at the cathodes surface is certainly the creation of a SEI that formed on the LNMO cathode.

4-(Trifluoromethyl)-benzonitrile (4-TB) was used as an electrolyte additive for LNMO cathode by Huang et al. [180]. Charge–discharge tests showed that the cyclic stability of LNMO was significantly improved by using 0.5 wt% 4-TB. With using 4-TB, LNMO delivered an initial capacity of 133 mAh g^−1^ and maintained 121 mAh g^−1^ after 300 cycles with a capacity retention of 91%, compared to the 75% of that using base electrolyte (1 M LiPF_6_ in EC/DMC). The results from linear sweep voltammetry, density functional theory calculations, electrochemical impedance spectroscopy (EIS), SEM, energy dispersive spectroscopy (EDS), FTIR, and inductively coupled plasma indicated that 4-TB had lower oxidative stability than EC and DMC, and was preferentially oxidized on LNMO forming a low-impedance protective film, which prevented the subsequent oxidation decomposition of the electrolyte and suppressed the manganese dissolution from LNMO.

Electrolytes modifying have similar effects with cathode surface coating on the cycling stability of LNMO cathodes. The working mechanisms of electrolytes modifying are preventing electrode reaction with the electrolyte and protecting cathodes from crystal destruction through inhibiting the electrolyte decomposition at the electrode/electrolyte interface. Therefore, the structure integrity and capacity retention during charge/discharge cycles are improved by electrolytes modifying. Compared with cathode surface coating, solid electrolytes and fluorinated electrolytes directly shield the undesired reactions between electrolyte and electrode due to the fact that solid electrolytes do not have fluid solvents and fluorinated molecules have higher oxidation potentials than their non-fluorinated counterparts. Electrolyte additives could produce in situ self-formed SEI films on the surface of LNMO cathodes in LIBs during the first charge and discharge, and the thickness of SEI films could be controlled by adding different concentrations of additive in the electrolyte. Therefore, electrolyte modifying is the most effective and easiest strategy to enhance the cycling stability of LNMO cathodes and easy to implement industrial production.

### Others

In addition to doping, cathode surface coating, and electrolyte modifying, there are some other technologies used for improving the cycling stability of LNMO cathodes. Deng et al. [33] synthesized double-shell LNMO hollow microspheres via a facile molten salt and annealing method. When applied as cathode materials for HVLIBs, the capacity of double-shell LNMO hollow microspheres remained about 98.3% after 100 cycles (116.7 mAh g^−1^ at 0.5 C between 3.5 and 5.0 V) because the double-shell structures allowed easy penetration of the electrolyte into the whole microspheres and buffer the large volume change of the electrode materials during Li-ion intercalation/deintercalation processes. One-dimensional porous nanostructures of LNMO were obtained through solid-state Li and Ni implantation of porous Mn_2_O_3_ nanorods that resulted from thermal decomposition of the chain-like MnC_2_O_4_ precursor by Zhang et al. [181]. The fabricated LNMO delivered specific capacities of 140 and 109 mAh g^−1^ at 1 and 20 C rates, respectively. At a 5 C cycling rate, a capacity retention of 91% was sustained after 500 cycles, with extremely low capacity fade (<1%) during the initial 300 cycles. The remarkable performance was attributed to the porous 1D nanostructures that accommodated strain relaxation by slippage at the subunits wall boundaries and provided short Li-ion diffusion distance along the confined dimension. Zhou et al. [182] synthesized uniform LNMO hollow microspheres/microcubes with nanosized building blocks by a facile impregnation approach. The resultant LNMO hollow structures delivered a discharge capacity of about 120 mAh g^−1^ with excellent cycling stability, which might be attributed to the unique nano/micro-hierarchical structure. Specifically, the nanosized/submicrometer-sized building blocks provided short distances for Li^+^ diffusion and large electrode–electrolyte contact area for high Li^+^ flux across the interface. Second, the structural strain and volume change associated with the repeated Li^+^ insertion/extraction processes could be buffered effectively by the porosity in the wall and interior void space, thus improving the cycling stability. However, these methods were not systematically studied because they did not fundamentally prevent electrode/electrolyte interface degradation, gas production, transition metal dissolution, and other undesired reactions between electrolyte and electrode during high-voltage cycle.

## Remarks and Conclusions

Compared with improving the discharge specific capacity and enhancing the platform voltage, the latter is easier to reach the prospective ED of LNMO for the practical application. Unfortunately, the cycling degradation of LNMO at high voltage becomes the biggest limit in application. Against the disadvantages, lots of studies found that electrode/electrolyte interface degradation, gas production, and transition metal dissolution are the main factors in cycling degradation; the essence of these factors is undesired side reactions between the electrode and the electrolyte. As well as, LNMO with the space group of $Fd\bar{3}m$ has superior electrochemical behavior and structural reversibility compared to *P*4_3_32. On this basis, various kinds of strategies were used to reduce cycling degradation, which could be summarized as doping, cathode surface coating, electrolyte modifying, and other effective methods. Doping improved the cycle performance of LNMO mainly via metal ion changing the crystal compositions, structures, and parameters, as well as promoting the formation of $Fd\bar{3}m$ structure. However, it is not able to prevent the undesired side reactions between cathode and electrolyte. Cathode surface coating could effectively prevent the undesired side reactions by using the coating layer on the surface of LNMO, but the coated technology is complex under normal conditions. Electrolyte modifying is an ideal strategy compared with doping and cathode surface coating; it not only prevents the undesired side reactions between cathode and electrolyte but also possesses easy technology. Although other methods also could improve the cycle stable of LNMO in HVLIBs, they are not able to stop the undesired side reactions.

In summary, recent studies have demonstrated that LNMO is a potential cathode material for HVLIBs, especially, the LNMO crystal with the space group of $Fd\bar{3}m$. According to study, enhancing the Platform voltage is a good method to improve ED of LNMO. Doping, cathode surface coating, and electrolyte modifying are able to reach the desired cycling stability in HVLIBs. In this paper, we summarized these approaches to improve the cycling stability of LNMO cathodes based on its architectural features and cyclic degradation mechanisms. However, there are still some challenges we should be faced. Firstly, cathode surface coating is difficult to extend for large-scale battery applications and reduces the discharge capacity of LNMO cathodes. Secondly, the ionic conductivity and electrode/electrolyte interfacial compatibility of solid electrolytes should be improved in the future. Thirdly, the relationships between LNMO structure (crystal parameter, particle morphology, crystal defect, etc.) and cycling degradation mechanism should be further studied. The investigations for high-voltage LNMO cathodes aim to find out the ways to improve cycle performances of LNMO and service for the life. With comprehensive research, we believe that LNMO will be widely used for the practical applications of high-power devices.

In the future, we should improve the cycle performance of LNMO based on the synthesis of highly purified LNMO, structural reversibility of $Fd\bar{3}m$, and cycling degradation mechanism of undesired reactions between LNMO and electrolyte. In various modification methods, organic coating and electrolyte additives may be good ways to improve the cycle performance of LNMO. However, considering the cost and practicality, electrolyte additives are superior to organic coating. Looking for the suitable electrolyte additives will be the next step work. In addition, other HVLIBs cathode materials also should be developed.
